# Translation and Validation of the Portuguese Version of European School for Interdisciplinary Tinnitus Research Screening Questionnaire (ESIT-SQ-PT)

**DOI:** 10.3390/audiolres16010002

**Published:** 2025-12-19

**Authors:** Haúla F. Haider, Ana Solange Fernandes, Ana Filipa Aguiar, Beatriz Oliveira, Iris Peixoto, Marília Antunes, Derek James Hoare, Helena Caria

**Affiliations:** 1 ENT Department, CUF Tejo Hospital, CUF Academic Center, 1350-352 Lisboa, Portugal; haula.f.haider@cuf.pt; 2 Comprehensive Health Research Centre, NOVA Medical School, Faculty of Medical Sciences, 1169-056 Lisbon, Portugal; 3 Biomedical Technology Bachelor, School of Technologies of Setubal, Polytechnic University of Setubal, 2914-503 Setúbal, Portugal; anasolange.inbox@gmail.com; 4 Biomedical Engineering and Biophysics Master, Faculty of Sciences, University of Lisbon, 1749-016 Lisbon, Portugal; 5 Biomedical Engineering Master, School of Health and School of Technologies of Setubal, Polytechnic University of Setubal, 2914-503 Setúbal, Portugal; ana.filipa.aguiar@estudantes.ips.pt (A.F.A.); iris.peixotor@estudantes.ips.pt (I.P.); 6 Biomedical Technology Bachelor, School of Health and School of Technologies of Setubal, Polytechnic University of Setubal, 2914-503 Setúbal, Portugal; beatriz.oliveira@live.com.pt; 7 CEAUL—Centro de Estatística e Aplicações, Faculdade de Ciências, Universidade de Lisboa, 1749-016 Lisboa, Portugal; mcreis@ciencias.ulisboa.pt; 8 Faculty of Sciences, University of Lisbon, 1749-016 Lisbon, Portugal; 9 National Institute for Health and Care Research (NIHR) Nottingham Biomedical Research Centre Sciences, Hearing Sciences, Mental Health and Clinical Neurosciences, School of Medicine, University of Nottingham, Nottingham NG7 2UH, UK; derek.hoare@nottingham.ac.uk; 10 Department of Speech and Hearing Sciences, University College Cork, T12 EK59 Cork, Ireland; 11 Biomedical Sciences Department, School of Health, Polytechnic University of Setubal, 2914-503 Setúbal, Portugal; 12 BioISI—Instituto de Biosistemas e Ciências Integrativas, Faculdade de Ciências, Universidade de Lisboa, 1749-016 Lisboa, Portugal

**Keywords:** tinnitus, hearing impairment measurement instrument, ESIT-SQ-PT, transcultural validation, psychometric characteristics

## Abstract

Objective: Several questionnaires for the diagnosis and characterization of tinnitus are available in English but there is a need for Portuguese standardized questionnaires for use in research and in clinic. The goals of this study were to translate and culturally adapt the ESIT-SQ (European School for Interdisciplinary Tinnitus Research Screening Questionnaire) to Portuguese, and to validate the questionnaire for clinical use. Methods: Translation and cross-cultural adaptation of the instrument were performed. The translation stage included the translation and retroversion of the instrument in the languages of interest (English–Portuguese) by three bilingual translators. Subsequently, cross-cultural adaptation was performed involving an Experts Panel (n = 5) and a Patient Panel (n = 4) to evaluate the questionnaire versions obtained after translation and retroversion. Participants completed their evaluation in Microsoft Forms. All ambiguities and uncertainties were addressed by the research team. Validation of the questionnaire involved an ENT specialist (n = 1), health researchers (n = 3), and patients (n = 300). Results: The Portuguese version of the ESIT-SQ (ESIT-SQ-PT) was found to be culturally appropriate, clear, and valid for clinical use. Expert review confirmed strong face validity, with only minor textual adjustments needed. The validation study, involving both online and paper responses, demonstrated good reproducibility and internal consistency across diverse participant profiles. The questionnaire effectively captured a wide range of tinnitus characteristics and associated factors, and reliability analyses confirmed its temporal stability. Overall, the ESIT-SQ-PT proved to be a robust and reliable instrument for assessing tinnitus in Portuguese-speaking populations. Conclusion: The ESIT-SQ in Portuguese (ESIT-SQ-PT), had good face validity, was comprehensible, and was culturally appropriate; thus, it is a valid tool for the screening and assessment of tinnitus and associated symptoms in Portuguese populations.

## 1. Introduction

Subjective tinnitus is the sensation of hearing a sound in the absence of an internal or external source. It is a common complaint encountered in primary care and can have a major negative impact on the quality of life of patients [1,2,3]. Tinnitus affects 750 million people globally, with more than 120 million identifying it as a major problem, most of whom are aged ≥ 65 years [4,5]. Etiology is frequently difficult to determine, and tinnitus is often described as being associated with hearing loss, deficits in attention and memory, and emotional stress [2]. There is no objective method for the assessment and diagnosis of tinnitus and questionnaires form a standard approach. The recently developed European School for Interdisciplinary Tinnitus Research Screening Questionnaire (ESIT-SQ) was conceived as a comprehensive tool for the global and standardized assessment of tinnitus patients and has already been translated into several languages [6,7,8]. However, translation, cross-cultural adaptation, and validation of this instrument into Portuguese is still outstanding.

The aims of this study were to (1) translate and culturally adapt the ESIT-SQ into Portuguese for both research use and clinical screening, following good practice guidelines for the translation of hearing-related questionnaires [9], and (2) test the validity of the ESIT-SQ in a large Portuguese-speaking (from Portugal) cohort, including the assessment of its psychometric properties.

To achieve these aims, the study followed a structured, multi-stage process that ensured methodological rigor and equivalence with the original English instrument [7]. It began with a rigorous forward–backward translation of the ESIT-SQ into European Portuguese to guarantee semantic and conceptual equivalence. This was followed by a cross-cultural adaptation phase, during which experts and patients evaluated the translated version for clarity, cultural relevance, and interpretability. The final stage involved psychometric validation to assess reliability, internal consistency, and convergent validity between ESIT-SQ and Tinnitus Handicap Inventory (THI) for some ESIT-SQ items that do provide a ‘measure’ [10]. This comprehensive process ensured that the ESIT-SQ-PT is not only linguistically accurate but also culturally appropriate and psychometrically robust for application in both clinical and research contexts within Portuguese-speaking populations.

## 2. Materials and Methods

### 2.1. The ESIT-SQ

The ESIT-SQ [7] consists of 56 closed-ended questions, mostly multiple choice. It consists of three parts: Part A, Part B, and an optional Part O. Part A includes 17 questions that can be answered by everyone, regardless of whether they have tinnitus or not. There are seven questions about demographics, body characteristics, education and lifestyle, one question about family history, and nine questions about medical history and presence of hearing-related and other symptoms. The final question in Part A asks about the presence of tinnitus lasting longer than 5 min over the past year. Participants who answer “yes” to this question are asked to complete Part B, an additional 22 questions related to tinnitus. These include eight questions about the perceptual characteristics of tinnitus, one general question about the impact of tinnitus, six questions about characteristics related to the onset of tinnitus, four questions about modulating factors of tinnitus and associations with coexisting conditions, one question about objective tinnitus, and two questions related to healthcare. Part O includes 17 questions that are extensions of the questions in Part A that ask for additional information about the participants’ lifestyle [6]. The ESIT-SQ was designed as a self-completion or “free of the clinic” tool, suitable for both screening and profiling of tinnitus.

### 2.2. Translation and Back-Translation

Translation and back-translation of the ESIT-SQ was performed in April and May 2021, and survey data were collected in May and June 2022.

Translation aimed to produce a Portuguese version, linguistically correct and equivalent to the original English version, rigorously in agreement with the good conduct guidelines for the translation of hearing-related questionnaires [7]. This step began with the production of two Portuguese versions, independently generated by two independent bilinguals (Portuguese–English) female translators. Both translators were researchers in genetic deafness and had lived in a country where English is the official language for more than 5 years. The two independent translations were compared by the research team, to obtain a single consensus version through the validation process abovementioned.

The consensus version obtained after the translation was subjected to back-translation into English allowing the comparison with the original version of the ESIT-SQ. Back-translation was performed by two bilingual female translators with Portuguese nationality and residence in England for over 20 years, and a bilingual translator with Philippine nationality, who lived in the Philippines for 16 years. The translated version of the questionnaire was assessed by the research team for accuracy of grammar and Portuguese language terms, and the back translated version was compared for parity with the original English version.

### 2.3. Cross-Cultural Validation

The preliminary version of the ESIT-SQ-PT was evaluated by an expert panel of four healthcare professionals in the field of Audiology or Otorhinolaryngology (three female, one male) and one female academic professional in the field of Audiology, each with more than 10 years of professional experience in this field. The evaluation was conducted using an electronic form (Microsoft Forms), where the pages of the questionnaire were divided into the three parts of the ESIT-SQ-PT—Part A, Part B, and Part O. They were asked to complete the questionnaire, and to screen for spelling or grammatical errors or problems in the formulation of the questions that might lead to ambiguity or difficulties in answering. In case of disagreement regarding a question, a text box was displayed, allowing for a proposal to change the sentence and a justification of the inadequacies of the question. Participant suggestions were reviewed by the research team to produce a consensus version. Following this, a test of comprehension and linguistic and cultural adequacy was performed with a small group of patients recruited from The ENT department of Hospital CUF Tejo and from the general population (a gender-balanced sample aged between 19–45 years old, studying in higher education or working in different fields presenting at least a bachelor’s degree) to finalize the adaptation stage. This test of content validity aimed to detect problems of ambiguity or difficulty in understanding by the patients. The Patient Panel was composed of four members (two male, two female), aged 30–40 years old, all with at least a bachelor’s degree. The questionnaire was administered online. During this test phase, some minor spelling and grammatical issues were identified, leading to some changes in wording to avoid ambiguity. The research team then approved this revised and final version of the questionnaire for validation.

### 2.4. Validation Study

Our inclusion criteria were being 18 years or older, knowing how to read and write, and having Portuguese nationality or a residence permission in Portugal. An exclusion criterion was the cognitive inability to complete an informed consent form and the questionnaires under study. The objective was to provide a sample of 100–300 participants, since this is typical for cross-validation studies [8].

Tinnitus (TIN) participants were recruited from the ENT department of the CUF Tejo Hospital (n = 98, 32.7%), and through word of mouth near the community of colleagues and friends (n = 202, 67.3%). This study was approved by the Ethical Committee and Personal Data Protection Departments of Hospital CUF Tejo (Declaration: #248).

Participants were informed about the purpose of the study and agreed to the terms and conditions. All participants completed the following documents: (1) Explanatory Letter and Informed Consent, (2) ESIT-SQ-PT, and in the case of participants with tinnitus, (3) the Tinnitus Handicap Inventory (THI) questionnaire [10]. The ESIT-SQ-PT questionnaire was completed at baseline (T0) and once again within 72 h (T1). While some of the T0 data were collected on paper (n = 97, 32.3%), all T1 data were submitted using an online version of the ESIT-SQ-PT and THI on the Microsoft Forms platform. This was due to the limitations of the participants regarding going to the hospital in the no tinnitus (STIN) group and returning to the hospital only to fill in the forms (in the TIN group).

A database was created using Excel files and data were coded to ensure anonymity and confidentiality.

Psychometric properties (validity and reliability) were assessed using Simple Concordance Coefficient (SCC) and the Spearman Correlation Coefficient (SP). SCC was selected for its capacity to assess both precision and accuracy and consider what provides a clear cut-off in terms of diagnostic accuracy (>0.9) which is useful in determining clinical value of this profiling questionnaire. For the latter (SP), a score was assigned to the ESIT-SQ-PT questions that had to fulfill two requirements: (1) to correspond with the THI questions, and (2) be sortable. Specifically, correlations were assessed between Question A13 from ESIT-SQ-PT and question F02 from THI, and question B4 from the ESIT-SQ-PT with questions E03, C05, E06, C08, F09, E10, F12, F13, E14, E16, F18, C19, F20, C23, and E25 of THI. This comparison between the ESIT-SQ-PT items and the THI validated in Portuguese from Portugal [10] tested convergent validity. This comparison was conducted solely to explore convergent tendencies, rather than to statistically validate the entire questionnaire. The primary validation of the ESIT-SQ-PT relied on qualitative assessments of face and content validity by both experts and non-experts, confirming its appropriateness and comprehensiveness for its intended clinical and research purposes.

Questions A13 and B4 of the ESIT-SQ-PT were scored on a scale of 0–4 and 0–3, respectively, with a score of 0 being assigned to the option “I have no difficulty” and “I don’t know” from question A13, and “Nothing” and “I don’t know” from question B4, increasing the score depending on the severity of the response (according to the number of options available in each question, which justifies the difference in the assigned scales). All calculations were performed using the RStudio software, version 2022.02.

As part of the study of the reliability of Part A of the ESIT-SQ-PT, there was a step to verify the agreement of questionnaire responses at T0 and T1.

To simplify data analysis, the 39 questions of the ESIT-SQ-PT instrument were divided into Parts A, B, and O. As such, the first division was assumed that it included questions related to the sociodemographic characteristics of the individuals belonging to the subsamples (A1-O6) and a second division that contained questions about the daily habits of the participants and their clinical history (O7-A17).

The scale for this coefficient varies between 0 and 1, being more consistent the closer to 1.

## 3. Results

### 3.1. Translation and Back-Translation

The results of the translation and back-translation process for the ESIT-SQ questionnaire yielded favorable outcomes, affirming the quality and suitability of the translated version. Thorough analysis of the consensus translation and back-translations showed minimal or negligible content discrepancies, validating the accuracy of the initial translation. Minor revisions were implemented to address linguistic and semantic disparities: a slight correction was made in question A13 of the questionnaire—change from “discriminar” (“distinguish”) to “ouvir” (“listen”)—and a revision of question B8, because there was a discrepancy in content regarding the original ESIT-SQ.

### 3.2. Cross-Cultural Validation

The evaluation conducted by the Experts Panel exhibited a consensus regarding the questionnaire’s content, with a limited number of suggestions pertaining to textual modifications. The Patients Panel provided insightful feedback, manifesting a greater tendency towards substantial modifications in question formulation compared to the Experts Panel. These suggestions were meticulously examined and integrated, adhering to predetermined criteria, ensuring grammatical precision and the incorporation of colloquial synonyms, while upholding the questionnaire’s original intent. These alterations aimed to improve the clarity, accuracy, and relevance of the questionnaire for its intended population.

Detailed records of Experts and Patients feedback, including examples of minor linguistic adjustments and the rationale for each modification, are provided in Supplementary Tables S1 and S2.

### 3.3. Validation Study

#### 3.3.1. Participant Characteristics

Ninety-seven participants (32.3%) completed the questionnaires on paper and 203 (67.7%) completed the questionnaires online. Sociodemographic data were obtained from participants in Part A of the ESIT-SQ-PT. We only considered data from the participants that had completed the questionnaires at both T0 and T1 since we wanted to study the reproducibility of the questionnaire.

The average age of TIN participants was 46.5 years (from 13 to 81 years). The STIN group has a mean age of 39.7 years, (from 18 to 66 years). Both groups had more females than other genders (A2), with 60% females in the STIN group (n = 106) and 52% in the TIN group (n = 75). There was no statistical difference between groups regarding sex distribution, height, or weight.

Most participants (58%, n = 175) had higher education. There were no differences between STIN and TIN individuals, regarding alcohol consumption or smoking habits. Regarding family history, the answer “not having any family member with tinnitus” represented 47.9% (n = 69) in the STIN group and 46.7% (n = 140) in the TIN group. In the STIN group, 63.5% (n = 99) reported not having vertigo compared to 41.7% (n = 60) in the TIN group. Having acute otitis was reported by 12.4% (n = 20) of STIN participants and 13.2% (n = 20) of TIN participants.

Most participants (66%, n = 95) who had tinnitus reported not experiencing discomfort from everyday sounds (hyperacusis) or difficulty hearing conversations in noisy environments (56.7%, n = 170). Most participants indicated having some difficulty hearing; however, only seven individuals used technical aids; 4.2% (n = 6) used hearing aids and one (0.7%) individual used a sound generator. Headache was the most reported pain syndrome; 25.6%, n = 51 of TIN individuals, and 21.3%, n = 38, of STIN individuals. An equivalent number of individuals in this sample has anxiety (10.8%, n = 32) (conditions diagnosed by a clinician).

It was observed that 91.6% (n= 274) of participants had right-hand dominance. Participants were mostly of Portuguese nationality with 99% of participants living in Portugal. Most participants were native Portuguese (90%, n = 270) and the remainder from Lusophone or European countries. Participants generally had an average economic condition (53.5%, n = 76) employed (52.1%, n = 75). Lifestyle patterns were typical for an adult population, with most respondents reporting 7 h of sleep per night, moderate physical activity (~2 h/week), and balanced dietary habits (fish and fruit 2–3 times/week).

Detailed sociodemographic, anthropometric, and lifestyle data—including distributions by age group, education level, comorbidities, and tinnitus history—are provided in Supplementary Tables S1 and S2.

#### 3.3.2. Tinnitus Characteristics

The majority (86.1%, n = 105) of the TIN participants reported having tinnitus daily or almost every day of their lives since its appearance (Question B1). Most participants (80.5%, n = 99) heard their tinnitus constantly or almost always under these circumstances and only 19.5% (n = 24) of individuals reported having intermittent tinnitus (Question B2). When asked how long ago their tinnitus appeared, 66 participants (54.1%) reported that they have had this symptom for years (average of 5.6 years since the first manifestation) (Question B3). Most individuals (70.5%, n = 93) described tinnitus as a single sound, and only 29.5% (n = 39) indicated hearing more than one different sound (Question B6).

Half of the participants (50%, n = 66) indicated that their tinnitus began suddenly and 26% (n = 34) indicated not knowing when tinnitus began (Question B7). Some participants associated the initial onset of tinnitus with an event specific (Question B9). This included stress (18.9%, n = 27) and “Other.” (21%, n = 30), where participants often indicated a relationship between tinnitus and COVID-19. Thirty-nine participants (31.0%) indicated that they were taking medication at the time of tinnitus onset (Question B10). Loud environments and stress were frequently identified as aggravating factors, while rest, relaxation, and adequate sleep were associated with reduced tinnitus perception.

Despite the presence of tinnitus, most participants (55.9%, n = 81) were not undergoing any type of treatment and most participants with tinnitus (75%, n = 91) did not consider having other conditions related to increased tinnitus (Questions B21 and B22). These characteristics are summarized in the table below (Table 1).

### 3.4. Psychometric Properties of the ESIT-SQ-PT

#### Validity

**Figure 1:**
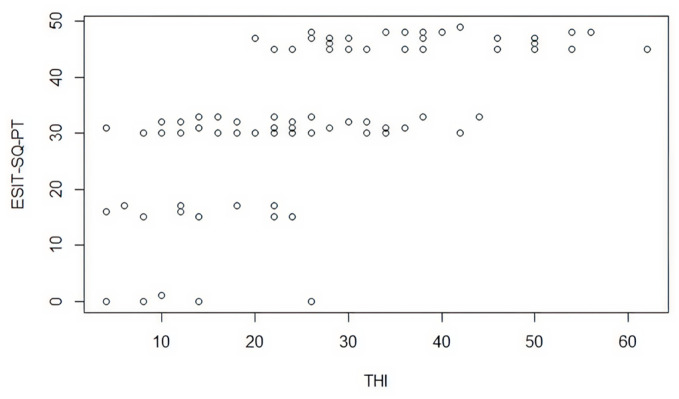
Results of the Spearman Correlation of the two instruments.

A score was assigned to the questions in the ESIT-SQ-PT questionnaire that corresponded to the questions in the THI questionnaire, which involves fifteen questions from the THI and only two from the ESIT-SQ-PT. Overall (Figure 1), there was a positive correlation between the items indicating they are measuring a similar construct. It is also important to notice that the mean THI score was 34 in a scale of 1–100 where 0–16 is slight or no handicap (Grade 1), 18–36 is mild handicap (Grade 2), 38–56 is moderate handicap (Grade 3), 58–76 is severe handicap (Grade 4) and 78–100 is catastrophic handicap (Grade 5), meaning that in this sample the mean indicated that there was a mild handicap (Grade 2) [10]. This group had a minimum of 0 in the THI questionnaire and a maximum of 96, which indicates a great range in the perception and handicap regarding the tinnitus.

##### Reliability Study

Reliability was quite satisfactory confirming that the questionnaire is functioning well, since it is quite stable in both groups, while still capturing normal changes that might occur associated with the different timepoints at which the questionnaire was completed; this likely reflects real variability in the tinnitus experience rather than measurement error. In addition to that, a high simple agreement coefficient means that there is a strong internal consistency and the questions are clear for the individuals [7], (Table 2).

Reliability of Part B of the ESIT-SQ-PT varied between 0.5 and 0.9, indicating some heterogeneity of responses, being the median greater than 0.5 (Table 3).

### 3.5. Portuguese-Language Version of the ESIT-SQ

The final Portuguese-language version of the ESIT-SQ is provided in Supplementary Materials, “Validated Portuguese translation of the ESIT-SQ questionnaire”. It is complete and includes all the optional fields of ESIT-SQ, in two parts, the first of which can be completed by anyone, whether experiencing tinnitus or not.

## 4. Discussion

The present study produced a validated Portuguese translation of the ESIT-SQ. We adhered to a rigorous translation procedure [10], ensuring linguistic accuracy and cultural relevance. The questionnaire content was piloted with four tinnitus patients before progressing to a further validation stage where validity was assessed.

The translations and back-translations processes, along with the suggestions of the Experts and Patients Panels, revealed a few discrepancies, which supplied confidence to the procedures. This suggests that the ESIT-SQ-PT can be reliably used in Portugal for tinnitus research, epidemiological surveys and in clinical practice. The tool may also be used in other Portuguese-speaking countries, with additional validation to check for regional linguistic differences.

While the translation and adaptation process was comprehensive, the validation study had certain limitations. First and foremost, psychometric validation remains preliminary; hence, further validation steps are needed, such as the use of larger samples or even clinical subgroups. Data were collected via a digital platform open to all-comers, with no supervision of response entries by a health professional. Responses were self-reported by platform users, who may have had problems with certain medical terms and in self-assessing medical criteria such as degree of hearing loss. Also, some items referred to past events, the memories of which could be defective.

Although the questionnaire was validated to some extent in reports of previous translations in other languages, it would still be useful to complete the validation by test–retest analysis and extensive psychometric tests. This would strengthen the claim of full validation and ensure its robustness for clinical applications.

Within the scope of this paper:

Part A

Regarding age, the average was lower for the STIN sample than the TIN sample, reflecting that the onset of tinnitus is associated with advancing age (A1) [11]. This finding supports the view that age-related auditory changes contribute to tinnitus onset, though further research is needed to clarify the mechanisms of this relationship. In terms of habits, while tobacco smoke and alcohol consumption are risk factors described for tinnitus, our data did not support the expected relationship between these factors and tinnitus, suggesting that other elements may be more significant contributors (A6 and A7) [12].

Hereditary tinnitus was not commonly observed in our sample (A8) [13], suggesting that genetic factors may not be the primary cause of tinnitus. This aligns with the multifactorial nature of tinnitus, where both genetic and environmental factors contribute to its onset.

Tinnitus was associated with vertigo in our studied sample (A9) [14]. The appearance of tinnitus can be indicative of the emergence of other diseases that affect the ears and auditory system, and, in our sample, acute otitis seems to be a factor associated with the appearance of tinnitus although the STIN and TIN responses are close to each other (A10).

We did not find a correlation between clinical interventions (e.g., chemotherapy, neurosurgery, etc.) and the onset of tinnitus (A11), suggesting that tinnitus may not be directly linked to medical treatments but could arise from the underlying conditions being treated.

Most of our participants with tinnitus did not feel discomfort in noisy environments and did not have trouble in hearing conversations (A12 and A13) [15]. This finding suggests that hyperacusis is not a universal feature of tinnitus, and its presence should not be assumed in all patients when planning treatment.

Although the sample included some participants with hearing difficulties, few used technical aids, which may be indicative of a lack of knowledge about the symptom or lack of financial resources (A14).

People who manifest tinnitus often have other comorbidities, such as headaches or neck pain but due to the similar results in subgroups STIN and TIN, we consider it is not a relevant factor for the onset of tinnitus (A15).

Both deviated nasal septum and anxiety were present in similar proportions in both the TIN and STIN groups (A16). While anxiety may exacerbate tinnitus perception, it does not appear to be a primary factor in tinnitus onset, suggesting the need for integrated psychological care in managing tinnitus.

Part O

Given that tinnitus can have different causes and there are neuronal pathways involved in its etiology, it is relevant to know which is the dominant hand of the participants although it does not seem to be considered a risk factor in our sample (O1) [3].

Lack of rest is often related to the appearance of tinnitus, due to its association with greater stress factors induced by sleep deprivation and situations of anxiety and even depression. However, in our study, night work and related sleep pattern changes did not appear to influence tinnitus occurrence (O7) [16,17]. This suggests that while sleep quality is important for overall well-being, its direct contribution to tinnitus onset may be less pronounced than previously thought.

Physical activity is described as a strategy used by individuals with tinnitus to compensate for the limitation caused by this condition, since physical effort causes a reduction in their perception; however, it does not seem to be a strategy used by the individuals in our TIN sample. The slightly higher value for physical activity in our STIN sample may be because they were younger individuals and currently the practice of exercise is highly valued among youth (O10) [18].

Healthy eating habits are crucial for healthy aging, and consequently, for a decrease in tinnitus. Despite this, there does not seem to be a relationship between these habits and the current onset of tinnitus (O11–O14) [18]. While diet alone may not directly influence tinnitus development, it remains an important factor in maintaining general health and potentially reducing the impact of comorbid conditions.

The use of a cellphone may be related to the appearance of tinnitus due to the sound impact it can have on the auditory system; however, a direct relationship between the hours of use of these devices and the onset of tinnitus was not observed (O15). Similarly, the use of headphones does not seem to be associated with the development of tinnitus (O16) [19].

Rest is essential for mental and physical health. Although sleep deprivation has been linked to tinnitus in other studies, in the current study there was no difference between TIN and STIN regarding the hours of sleep, thus it is not considered as relevant (O17) [20].

Part B

Tinnitus differs from individual to individual and can be perceived constantly in quiet environments or in an intermittent rhythm where the individual cannot always hear it. In our sample, the majority reported having tinnitus daily or almost every day. The symptom could occur constantly or almost always, with numerous factors that may be related to its development (B1 and B2) [21,22]. This emphasizes the chronic nature of tinnitus for most individuals and the need for long-term management strategies rather than short-term interventions.

Tinnitus may appear in childhood, or later, in adulthood, and may even occur in individuals who are already at a more advanced age (considered elderly) (B3) [11], with potential influences ranging from early noise exposure to age-related auditory decline.

Regarding the concern levels related to tinnitus (intrusiveness), almost half of participants felt only a moderate level of concern, which may be an indicative of a certain acceptance of the symptom by the individuals (B4). Sometimes having tinnitus is not synonymous with being bothered by it from when it first appeared.

Although some people have only one type of tinnitus, there are cases in which tinnitus can differ and be perceived in diverse ways, often represented as a single sound or more than one different sound [11]. Most TIN participants described their tinnitus as being a single sound and only some participants reported hearing two or more different sounds (B6).

The onset of tinnitus manifestation is remarkably diverse and may be gradual or sudden [21,22]. In our sample, half of participants indicated that the symptom appeared suddenly, and some did not know when their tinnitus appeared (B7). It is possible that the onset of tinnitus is associated with a relevant event in the life of the individual. However, in our sample there was no relationship between the proposed options of events and the appearance of the tinnitus in the participants’ lives. Despite this, stress appears to be described as a possible trigger for tinnitus onset throughout the testimonies collected from participants (B9); there was a strong tendency to associate the appearance of tinnitus with COVID-19 and the indication that they were not taking any type of medication (B10). This suggests that taking medication may not be relevant to tinnitus onset. Nevertheless, there are more than 200 drugs described as facilitators of tinnitus onset or worsening [21,22]. This discrepancy highlights the need for controlled studies to clarify the pharmacological influence on tinnitus development.

Since frequently other diseases may be related to the appearance of tinnitus, it is worth noting the importance of asking participants if they perceived this relationship. Responses did not suggest a direct relationship between past illnesses and the occurrence of tinnitus (B11) [11], indicating that tinnitus may arise independently of major medical conditions in many individuals.

Tinnitus can differ greatly in terms of intensity and may be stable over time or present variations [11]. Within our sample, participants considered their symptoms variable and described them as a single tone (B12 and B13). The pitch of tinnitus can differ greatly from person to person and can be characterized as having a high pitch, medium pitch or low pitch [11]. Some participants in the current study indicated that their tinnitus was loud and heard in both ears (B14 and B15), though without any apparent rhythmicity (B16). In most cases, tinnitus episodes are only heard by the individual affected. However, in rare cases, it can be heard by others [11]. Despite the rarity of the occurrence of these cases, there were reports from participants who indicated that they had already suffered from episodes of tinnitus audible by other individuals (B17), supporting the existence of uncommon objective tinnitus, though this remains exceptional.

Generally, there is no method that reduces the intensity or even the frequency of tinnitus. However, it is possible to reduce perception. To this end, indications have emerged that superior quality sleep, being relaxed, and being in the presence of moderately loud sounds are factors that help reduce tinnitus perception and therefore it is believed that those aspects are generally recommended for tinnitus patients (B18). Other factors increase its perception, such as being in a silent environment and being stressed or anxious have proved examples so these should be avoided (B19).

Seeking medical help is a crucial factor for accepting tinnitus. At the same time, it is important that patients learn how to self-manage it. Although our participants indicated resorting to specialized medical help, there were reports of participants who did not seek any type of specialized medical help. This may be indicative of the individuals financial/temporal unavailability, or the lack of information regarding tinnitus whereby it is not considered a priority to address (B20). Finally, despite stress being considered a factor that greatly influences lifestyle and its tinnitus, our participants did not report this association (B21 and B22), suggesting that individuals may not always recognize or attribute psychological factors to their tinnitus experience.

### 4.1. Validity of the ESIT-SQ-PT

As shown in Figure 1, four distinct levels of responses are observed. It is considered that this occurs due to the dispersion of the THI questions that correspond exactly to the same question of the ESIT-SQ-PT instrument, which explains the lack of a homogeneous dispersion that attributes a correlation between both instruments.

However, the existence of levels shows that within them, there is a correspondence between the questions of both instruments even if it does not seem to be possible to validate the ESIT-SQ-PT with the THI.

Previous validation studies of the ESIT-SQ [6,7,8] applied distinct methodologies to assess its validity. Some studies focused on linguistic and cultural validation of the French version through expert review and pilot testing, without statistical construct analysis [6]. Other studies used exploratory and confirmatory factor analysis to validate the questionnaire’s internal structure and demonstrated convergent validity with clinical measures [7]. There were even some studies that applied ESIT-SQ clinically to patients with chronic tinnitus, confirming content suitability without external correlations [8]. In contrast, the present study combined content validation—involving experts and patients—with convergent validation using Spearman’s correlation between ESIT-SQ-PT and THI items. Spearman’s method was appropriate due to the ordinal nature of the data. Despite the methodological differences across studies, all yielded satisfactory results, reinforcing the validity of the ESIT-SQ in diverse linguistic and clinical contexts.

### 4.2. Reliability of the ESIT-SQ-PT

Considering all the results presented, is possible to accept that the questionnaire is reliable and validated in the Portuguese language. The agreement between T0 and T1 shows some variation but this does limit the validation of the questionnaire.

It should also be considered that the perception of tinnitus in everyday life varies according to its manifestation and, therefore, we should consider the manifestation of the symptom on the day that the participants answered the questionnaire, which may influence their responses.

The development of ESIT-SQ-PT would contribute to improve any Tinnitus Database by sharing Portuguese data, ensuring the advantage of the standardization screening in clinical settings.

Previous studies testing the reliability of ESIT-SQ [6,7,8] used different approaches to assess this, all yielding satisfactory outcomes. There was a study in which it was confirmed with strong internal consistency, in this case in the French version of the questionnaire, despite focusing primarily on linguistic adaptation [6]. Then, there were reports of high internal consistency and temporal stability through Cronbach’s alpha and test–retest analysis [7]. Finally, a study [8] demonstrated consistent response patterns, particularly in Spanish patients with chronic tinnitus, supporting clinical reliability. In the present study, reliability was assessed via test–retest agreement and internal consistency, with coefficients ranging from 0.5 to 0.9 and a high simple agreement rate. These results confirm that, despite methodological differences, the ESIT-SQ-PT is a reliable tool for tinnitus assessment in the Portuguese population.

## 5. Study Limitations

The tinnitus group had a minimum score of 0 in the THI questionnaire and a maximum of 96 (mean 34), revealing a great variability regarding the tinnitus handicap. We consider that if our TIN population were more severely affected, the application of ESIT-TQ could have yield other results.

## 6. Conclusions

The ESIT-SQ-PT (Annex 1) showed good face and content validity, was comprehensible, and was culturally appropriate, confirming that it is a valid tool for the screening and assessment of tinnitus and associated symptoms in Portuguese populations. In addition, the test–retest agreement, internal consistency measures, and the convergent validity observed with THI items further support its reliability and psychometric adequacy. Therefore, the ESIT-SQ-PT will enable robust epidemiological and clinical data collection within Portuguese populations and allow meaningful comparison with datasets obtained in other countries using the corresponding validated ESIT-SQ versions.

## Figures and Tables

**Table 1:** Tinnitus characteristics.

Characteristics		%(n)
Tinnitus daily or almost every day		86.1% (105)
Heard their tinnitus constantly or almost always		80.5% (99)
Having intermittent tinnitus		19.5% (24)
Having tinnitus for years		54.1% (66)
Moderate level of concern regarding tinnitus		41.7% (55)
Feeling bothered for some time		47.9% (58)
Tinnitus as a single sound		70.5% (93)
Tinnitus began suddenly		50% (66)
Not knowing when tinnitus began		26% (34)
Initial onset of tinnitus related with stress		18.9% (27)
Initial onset of tinnitus related to another event		21% (30)
Taking medication at the time of tinnitus onset		31.0% (39)
No direct relationship between the disease and the onset of tinnitus		71% (93)
Tinnitus loudness variable		54.2% (71)
Tinnitus as a tone		37.5% (51)
High pitched tinnitus		43.3% (55)
Bilateral tinnitus		23.3% (31)
Tinnitus not rhythmic		96.6% (98)
Tinnitus audible by another person		24.6% (32)
Reduction in tinnitus	Optimal quality of sleep	13.2% (26)
	Being relaxed	11.2% (22)
	Loud sounds	18.8% (37)
	Nothing	17.8% (35)
Increasing in tinnitus	Silent environment	31.1% (70)
	Being stressed or anxious	18.7% (42)

**Table 2:** Simple agreement coefficient results for the STIN and TIN groups in the ESIT-SQ-PT Part A and Part O).

		Min. ^1^	1st Qu. ^2^	Median	Mean	3rd Qu. ^3^	Max ^4^
A1-O6	STIN	1	1	1	1	1	1
A1-O6	TIN	0	0	0.82	0.54	0.91	0.91
O7-A17	STIN	0.91	1	1	0.99	1	1
O7-A17	TIN	0	0.03	0.83	0.56	0.91	1

^1^ Minimum; ^2^ 1st quartile; ^3^ 3rd quartile; ^4^ maximum; TIN—tinnitus participants; STIN—non-tinnitus participants.

**Table 3:** Simple agreement coefficient results for the TIN group (Part B).

		Min. ^1^	1st Qu. ^2^	Median	Mean	3rd Qu. ^3^	Max ^4^
B1-B22.1	TIN	0.03	0.09	0.63	0.49	0.83	0.94

^1^ Minimum; ^2^ 1st quartile; ^3^ 3rd quartile; ^4^ maximum; TIN—tinnitus participants.

## Data Availability

The data presented in this study are openly available in [Polytechnic University Scientific Repository] [https://bibliotecas.ips.pt/servicos/repositorio-cientifico].

## Supplementary Materials

The following supporting information can be downloaded at: https://www.mdpi.com/article/10.3390/audiolres16010002/s1, Table S1: Sociodemographic, Anthropometric, and Lifestyle Characteristics of Participants; Table S2: Comparison of STIN and TIN groups.

## Abbreviations

The following abbreviations are used in this manuscript:

TIN	Tinnitus group
STIN	No tinnitus group
ESIT-SQ-PT	European School for Interdisciplinary Tinnitus Research Screening Questionnaire—Portuguese version
