# Supplementary material for: Translation and Validation of the Portuguese Version of European School for Interdisciplinary Tinnitus Research Screening Questionnaire (ESIT-SQ-PT)
Source: Audiol Res. 2025 Dec 19;16(1):2. doi: 10.3390/audiolres16010002 (PMC12821391; doi:10.3390/audiolres16010002)
Supplement: Supplementary file 1 [file audiolres-16-00002-s001.zip › audiolres-3934612-supplementary.pdf]

Translation and validation of the Portuguese version of European School for Interdisciplinary Tinnitus Research Screening Questionnaire (ESIT-SQ-PT)

SUPLEMENTARY MATERIALS

1. Detailed records of Experts and Patients feedback, including examples of minor linguistic adjustments and the rationale for each modification

In Part A of the questionnaire, the following modifications were made:

Question A2 - An additional option, "Other," was included to provide respondents with a broader choice selection.

Question A5 - The sentence "mais graus" was replaced with "superior" to ensure a more precise description of the intended meaning.

Question A6 - The statement was amended to include the unit of measurement "mL per beverage," providing a clearer reference for respondents.

Question A9 - The term "vertigens" (vertigo) was changed to "vertigem" for improved accuracy. Additionally, the phrase "rotação" (rotation) was modified to "andar à roda" to enhance understanding.

Question A10 - The word "criações" was replaced with "variações" to better capture the intended concept.

Question A11 - The term "intervenções" (interventions) was adjusted to "tratamento" (treatment) to accurately reflect the nature of the options presented.

Question A13 - The sentence "ouvir conversas" was revised to "entender conversas" to ensure a more precise descrip-tion of the desired ability.

Question A17 - The term "zumbidos" was changed to "zumbido" to align with the singular form of the phenomenon being addressed.

In Part B of the questionnaire, the following modifications were made:
Question B1 - The phrase was modified to include "os seus" for a more comprehensive understanding of the subject matter.
Question B4 - The word "Pouco" was replaced with "Ligeiramente" to convey a more accurate intensity level.
Question B8 - The phrase "ao lado" was adjusted to "abaixo" to better reflect the intended direction.
Question B10 - An explanatory note was added to define “analgésicos” as "(medicamentos para as dores)" for better clarity.
Question B17 - The term "médico" (medical doctor) was replaced with "profissional de saúde" (heath professional) to encompass a broader range of healthcare providers. Questions B18 and B19 - The phrase "a intensidade" was added to provide additional context for respondents.
Question B20 - The statement was modified to include "ou outro profissional de saúde" to acknowledge alternative healthcare professionals. Question B21 - The preposition "de" was inserted before "fisioterapia" to ensure grammatical accuracy.

In the Optional section of the questionnaire, Part O, the following modifications were made:
Question O4 - The options "Casado" and "União de facto" were combined to facilitate a more streamlined response process
Question O6 - The term "Empregado" was replaced with "Trabalhador por conta de outrem" to provide a more precise employment category.

2. The Patients Panel provided insightful feedback, manifesting a greater tendency towards substantial modifications in question formulation compared to the expert panel. Nevertheless, their contributions were thoughtfully incorporated, concentrating on grammatical enhancements and the incorporation of colloquial synonyms.
The changes made in Part A were "Lupus sistémico eritematoso" to "Lupus eritematoso sistémico" (Question A16).
In Part B, the adjustments consisted of changing the format from "__meses __anos" to "__anos __meses" (Question B3), rephrasing "seguintes perguntas" to "perguntas

seguintes" (Question B6), replacing "Gradual" with "Gradualmente" and "Repentino" with "Repentinamente" (Question B7), adding the option "10. Não sei" (Question B9), refining "9. Não" to "9. Nenhum medicamento" (Question B10), replacing "foram ouvidos" with "foram analisados" (Question B17), changing "reduzem a" to "di-minuem a" (Question B18), replacing "aumentam a" with "aumentam de" (Question B19).

In the Optional Part O, the following modifications were made including using the feminine forms of certain words (Question O4) and modifying "Não utilizo" to "Não uso" (Question O16).

These edits provided clarity, comprehensibility, and linguistic fidelity of the questionnaire, while preserving the inherent meaning of the questions

3. Supplementary Tables

Sociodemographic, anthropometric, and lifestyle data are provided in Tables below:

Supplementary Table S1– Sociodemographic, Anthropometric, and Lifestyle Characteristics of Participants

Variable	Total Sample	Observations
Sex distribution, height, weight	No statistical difference between groups	—
Education level	58% (n=175) with higher education	—
Alcohol consumption / Smoking habits	No difference between groups	—
Hyperacusis (discomfort from everyday sounds)	66% (n=95) reported no discomfort	Applies to tinnitus participants
Difficulty hearing in noisy environments	56.7% (n=170) reported no difficulty	—
Use of hearing aids / sound generator	4.2% (n=6) used hearing aids; 0.7% (n=1) used sound generator	—
Clinically diagnosed anxiety	10.8% (n=32)	—
Hand dominance	91.6% (n=274) right-handed	—
Nationality	99% living in Portugal	90% native Portuguese; remainder Lusophone/European

Economic condition	53.5% (n=76) average conditions	—
Employment status	52.1% (n=75) employed	—
Sleep duration	~7 hours/night	—
Physical activity	Moderate (~2 h/week)	—
Dietary habits	Fish and fruit 2–3 times/week	—

Supplementary Table S2– Comparison of STIN and TIN groups.

Variable	STIN Group	TIN Group
Family history of tinnitus	47.9% (n=69) reported no family member with tinnitus	46.7% (n=140) reported no family member with tinnitus
Vertigo	63.5% (n=99) reported no vertigo	41.7% (n=60) reported no vertigo
Acute otitis history	12.4% (n=20)	13.2% (n=20)
Headache (pain syndrome)	21.3% (n=38)	25.6% (n=51)
Work during the night	62,2 % (n=97) never work	67,1% (n=96) never work
